# The Pathology of Carcinoma

**Published:** 1894-12-15

**Authors:** 


### Progress in Surgery.

### THE PATHOLOGY OF CARCINOMA.

In The Hospital for June 30th we referred to a paper by Dr. Adler on the present position of our knowledge of protozoa and carcinoma. Since the publication of this paper the subject has been much discussed, and was one of the topics for debate at the recent International Medical Congress.[1] Professor Pio Foà, of Turin, and Dr. Armand Ruffer maintained that protozoa were present, whilst Professor V. Cornil, of Paris, disputed such a conclusion, and referred all the bodies described as protozoa to changes in the cell and nuclei. Both Foà and Ruffer rejected the bodies described by Dairer, Wickham, Podwysozki, Sawtschenko, Korotneff, and others as simply invaginated cells or products of degeneration, but maintained that the bodies they described as protozoa essentially differed from such. Dr. Ruffer considered that he had seen leucocytes penetrate into the epithelial cells in cancer, &c., carrying off the parasites which they found there. He was at present engaged in studying fresh preparations of cancer mounted in cancer juice, and he had observed movements of the protozoa.

Kanthack[2] considers that workers all through have neglected to examine ordinary tissues and ordinary non-cancerous processes as critically and minutely as they have searched malignant growths. He has found typical so-called cancer parasites staining exactly like Ruffer's with the Ehrlich-Biondi stain in a case of non-malignant sero-cystic disease of the breast, which contained peculiar giant cells formed by fusion of epithelial cells, and also bodies, so far as he could judge, identical with some of the protozoa described by Ruffer in ordinary blisters.

Kahane has gone a step further than other investigators, and has announced[3] the discovery of amæboid bodies, with very active movements, in the blood of patients suffering from carcinoma. They were found free in the blood stream, and also within the corpuscles, and the same bodies were discovered in the cells of the growths.

The question of the possibility of inoculating cancer has been lately discussed by Mr. Shattock in the Morton lecture on cancer,[4] and at the International Medical Congress. Mr. Shattock and Professor Foà[5] consider there is evidence that cancer has been communicated from one animal to another of the same species in this way. Professor Trasbot[6] doubted this. He had personally made numerous inoculations of canine carcinoma into dogs, and had only once obtained a positive result, and in that case the tumour only grew for a few months, and was then reabsorbed without any metastasis forming in internal organs. Although cancer was common enough in dogs, he thought tubercle had many times been mistaken for cancer, and hence the so-called successful inoculations of cancer in dogs must be regarded with the greatest scepticism, unless controlled by an accurate histological examination. Dr. Cazin[7] had also failed in his attempts to inoculate animals of the same species with carcinoma, although he had always been successful in grafting the growth on the same animal. He had, however, been successful in grafting three non-malignant growths into other animals. Dr. Ruffer believed that in order to obtain positive results it would be necessary to inoculate the metastasis, and not the primary tumour. Dr. Boinet[8] has inoculated cancer from human beings into animals sixty times. The only success obtained was from one intraperitoneal graft out of forty, one subcutaneous injection of the juice of a "lympho-sarcoma" of the testis into the subcutaneous tissue, and one of the same fluid into the pleura. Fifteen subcutaneous inoculations of cancer were negative in their result. All attempts to inoculate glands, such as the breast or testicle, failed. The one successful case of intraperitoneal inoculation is of great interest. A portion of epithelioma of the penis was placed in the peritoneum of a rat. Five weeks later the animal died, after suffering from paraplegia. Two fragments of growth were found in the peritoneum, and some nodules in the liver. The bodies of the vertebræ were also affected by the growth, and the cord had become compressed.

Of course the successful result of the inoculation of cancer does not in any way prove its parasitic origin. In order to do so it would be necessary to isolate by culture the protozoa, or to make sure the tissue cells of the graft were dead. Up to the present time all attempts to cultivate any organism from carcinoma have failed. Mr. Shattock[9] has been lately encouraged in his hope of being able to cultivate such micro-organism. He has obtained actively moving amæbæ from sterilised sand infected by carcinomatous growths.

Mr. Shattock[10] in the Morton lecture on cancer has lately called attention to the fact that several persons living in the same house, one after another, have developed cancer and died. He refers to one case described by Mr. Webb. In a small village in Shropshire were two houses under one roof. In one house a man died of cancer of the rectum; the next occupiers died, one of cancer of the rectum the other of cancer of the stomach. Then the house was taken by three spinsters. One died from cancer of the uterus, another from cancer of the stomach. Mr. D'Arcy Power[11] mentions several other instances almost as striking. They are not given as examples of the contagiousness of cancer, but rather of its endemic character in certain districts. Dr. Haviland concludes from the statistics of cancer he has collected that cancer is most prevalent along the courses of rivers which seasonally flood their banks, and especially where, from the flatness of the country, the floods were retained. In a more recent communication[12] read in the Section of Pathology at the last annual meeting of the British Medical Association, Mr. D'arcy Power related several experiments he has lately tried in keeping rats in cages over earth removed from low-lying districts near the rivers after a heavy rainfall, when the floods had recently subsided, in districts which had an evil reputation according to the statistics of Mr. Haviland on account of the frequent occurrence of cancer in them. Such districts were the valley of the Warwickshire Avon, the Thames at Oxford, the Ouse at Bedford, and the Kennet at Maldon, in Yorkshire. It was not supposed that the soil from these places contained the cancer germs, but that they might furnish a suitable medium for their growth and development, so that animals might become infected from cancer particles mixed with such soil. Portions of cancerous growths were minced up with normal saline solution and sprayed over the soil. The mucous membrane of the rats was also kept in a condition of chronic irritation. The experiment was continued for eleven months, but only with negative results.

[1] Med. Week, April 6, 1894, p. 160; Lancet, April 14, 1894, p. 958; Brit. Med. Journ, April 7, 1894, p. 743; N.Y. Medical Record, May 5, 1894, p. 567. [2] Med. Chronicle, April, 1894, p. 40. [3] Centralbl. f. Bakt. B. XV. No. 12; and Brit. Med. Journ, May, 26, 1894, p. 84. [4] Brit. Med. Journal, 1894, vol. i., p. 1065 [5] Brit. Med. Journal, 1894, vol. i., p. 743. [6] Ibid. [7] Ibid. [8] Medical Week, June 15, 1894, p. 285. [9] Brit. Med. Journ, 1894, vol. i., p. 1065. [10] Ibid. [11] Brit. Med. Journal, June 9, 1894, p. 1240. [12] Brit Med. Journ., Sept. 22, 1894.

